# The Use of Molecular Biology Methods to Evaluate the Activity of Different Topical Treatments Against Periodontal Pathogen Bacteria

**DOI:** 10.3390/pathogens15020197

**Published:** 2026-02-10

**Authors:** Alessia Pardo, Salma Hamzaoui, Annarita Signoriello, Elena Messina, Maria del Mar Lleò, Gloria Burlacchini, Andrea Butera, Andrea Scribante, Giorgio Lombardo, Caterina Signoretto

**Affiliations:** 1 Dentistry and Maxillo-Facial Surgery Unit, Department of Surgery, Dentistry, Pediatrics and Gynecology (DIPSCOMI), University of Verona, Piazzale L.A. Scuro 10, 37134 Verona, Italy; alessia.pardo@univr.it (A.P.); annarita.signoriello@univr.it (A.S.); giorgio.lombardo@univr.it (G.L.); 2 Diagnostic and Public Health Department, University of Verona, 37134 Verona, Italy; hamzaoui.s0079@gmail.com (S.H.); maria.lleo@univr.it (M.d.M.L.); gloria.burlacchini@univr.it (G.B.); caterina.signoretto@univr.it (C.S.); 3 Unit of Orthodontics and Pediatric Dentistry, Section of Dentistry, Department of Clinical, Surgical, Diagnostic and Pediatric Sciences, University of Pavia, 27100 Pavia, Italy; andrea.scribante@unipv.it

**Keywords:** periodontal pathogens, bacteria, periodontal disease, desiccant agent

## Abstract

Background: Periodontal disease results from a complex interaction between the microbial biofilm and the host immune response. The aim of this study was to evaluate and compare, in samples of dental plaque in periodontal patients, the presence of periodontal bacteria before and after two different non-surgical treatments: ozone (O_3_) therapy and a desiccant agent (HybenX, HBX, administered one or three times). Methods: Molecular biology techniques were used to estimate the effect of the two treatments on different periodontal pathogen microorganisms. The presence of *Porphyromonas gingivalis*, *Treponema denticola*, *Prevotella intermedia*, *Tannerella forsythia*, *Actinomyces naeslundii* and *Aggregatibacter actinomycetemcomitans* was investigated by multiplex PCR (mPCR) and quantitative PCR (qPCR) at baseline (T = 0, before oral hygiene), one week (T = 1), two weeks (T = 2), one month (T = 3) and three months (T = 4) after treatment. Results: *P. intermedia* was the most frequently detected pathogen in the study population, further quantified by qPCR in samples positive to mPCR at baseline (T = 0) and at the end of treatment (T = 4). The qPCR results showed evident decreases in load after treatment with HBX ^x^1, HBX ^x^3 and O_3_; nevertheless, comparison between groups and between time points (from T = 0 to T = 4) did not show any significant differences (*p* = 0.3 and *p* = 0.8). For *P. gingivalis*, the O_3_ therapy showed a reduction in detection after two weeks and after one month, while HBX showed a great reduction in its presence when administered three times. Conclusion: Both agents were effective in reducing the presence of the periodontal pathogens in the dental pockets of patients affected by chronic periodontal diseases. In particular, HBX applied three times showed greater improvement compared to a single application.

## 1. Introduction

The human oral cavity harbors a complex microbiota consisting of over 700 different microbial species (including bacteria, fungi, viruses, and protozoa), which play an essential role in maintaining balanced functions of nutrients’ metabolism, protection against external pathogens and regulation of the immune system [1].

This oral microbiota begins to establish itself shortly after birth and, continuing to evolve throughout an individual’s lifetime, it is influenced by diet, oral hygiene practices, and environmental exposures. A disruption in the equilibrium, known as dysbiosis [2], can thus lead to the development of gingivitis and periodontitis, which represent chronic inflammatory conditions affecting the soft and hard periodontal tissues around teeth, including gingiva, periodontal ligament, cementum and alveolar bone. Periodontal diseases represent a significant global health burden, and severe periodontitis is estimated to affect 10–15% of adults worldwide, being a major cause of tooth loss [3].

The primary cause of periodontitis can be identified in pathogenic bacteria in dental plaque biofilms, with specific roles of aggressive periodontal pathogens, including *Porphyromonas gingivalis*, *Tannerella forsythia* and *Treponema denticola*, which belong to the “red complex” of Socransky. Furthermore, periodontal diseases result from a complex interaction between this microbial biofilm and the host immune response, rather than from the action of a single pathogen alone [4,5].

In this proposal, the development of disease can be linked to genetic causes as well as the individual’s lifestyle [3], which favors the growth of pathogenic bacterial species. The clinical presentation typically begins with the accumulation of dental plaque, leading to gingivitis, a reversible inflammation of the gum. If left untreated, gingivitis can progress to periodontitis, characterized by the destruction of the periodontal ligament and alveolar bone loss [6], with increased progression in the presence of risk factors such as genetics, smoking, diabetes, and poor oral hygiene. It is thus fundamental to prevent or remove the formation of the biofilm [7] that makes up the dental plaque, which will turn into calculus after calcification.

On the other hand, it was also found that the composition of oral microorganisms is linked to systemic diseases [8,9,10,11], such as rheumatoid arthritis, septicemia, endocarditis, atherosclerosis, cardiovascular disease, diabetes, neurodegenerative conditions (Alzheimer’s disease), respiratory tract infections, and cancer (gastrointestinal, colorectal, and pancreatic cancers). This is probably due to the fact that these bacteria, predominantly Gram-negative, provide a high load of bacterial endotoxin (lipopolysaccharide-LPS) [12], which causes an inflammatory state even in other anatomical districts far from the oral cavity. Whenever the dysbiosis consequent to the accumulation of plaque is located, the prevalence of pathogenic species expressing their virulent factors leads to the development of the disease [13].

Understanding the complex microbial and host factors involved in periodontal diseases’ pathogenesis is crucial for developing effective prevention and treatment strategies to improve oral health. Given the increasing recognition of the links between oral health and various systemic diseases, this knowledge has implications not only for dental care but also for overall systemic health, and advancement in research perspectives is emerging for revolutionizing the approach to periodontal disease management and oral health promotion.

Consequently, current treatment approaches for periodontal diseases focus on non-surgical therapies, including mechanical debridement techniques, such as scaling and root planning [14], which aim to disrupt and remove pathogenic biofilms. The use of adjunctive antimicrobial treatments, both local and systemic, is also currently discussed for their benefits and potential drawbacks. The potential of emerging treatment options in overcoming limitations of traditional treatments, such as antimicrobial resistance, has been explored [15], including novel approaches like the HybenX (HBX) desiccant gel [16,17], which aims to dehydrate and disaggregate biofilms, facilitating biofilm removal, and the ozone–O_3_ therapy [18,19,20], which utilizes the antimicrobial properties of ozone gas. Few studies have also reported in vivo evaluation of periodontal bacteria following non-surgical treatments, based on the topical use of antibiotic gels [21,22] or with photodynamic therapy [23].

In this study, we aimed to evaluate and compare the in vivo effects of two antibiotic-free adjuvant treatments on the bacteria of dental plaque of subjects affected by periodontitis. Specifically, the antimicrobial activity of gaseous ozone (O_3_) and the desiccant agent HybenX (HBX) was compared in periodontal pockets of 30 patients affected by chronic periodontitis. The evaluation was performed using molecular techniques, such as multiplex PCR (mPCR) and quantitative real-time PCR (qPCR), detecting the main bacterial species responsible for the development and progression of periodontitis. Therefore, the aim of this study was to compare the presence of periodontal bacteria before and after the two different treatments, searching for the presence of *Porphyromonas gingivalis*, *Treponema denticola*, *Prevotella intermedia*, *Tannerella forsythia*, *Actinomyces naeslundii* and *Aggregatibacter actinomycetemcomitans*.

## 2. Materials and Methods

The microbiological investigation in this project was conducted at the microbiology laboratory of the University of Verona (Department of Diagnostics and Public Health), in collaboration with the Department of Dentistry and Maxillofacial Surgery of the Verona hospital, which performed the treatments and dental plaque sampling, and then clinically evaluated the efficacy of the two non-surgical adjuvant treatments in patients affected by periodontitis. This study was conducted between January 2024 and December 2024.

### 2.1. Inclusion Criteria for Patients and the Timeline of the Treatments

This study involved 120 samples from 30 patients with a diagnosis of stage II or stage III periodontitis (grades A to C) [24], in good general health, and without the performance of any periodontal treatment within 6 months from the enrolment date.

Exclusion criteria were:

− Pregnant and breastfeeding women;
− The absence of an assumption of antibiotic use;
− The absence of a smoking habit during the period of the treatment;
− No contraindications to periodontal treatments and the application of O_3_ and HBX.

The experimental protocol (Protocol HX-GL-ITA13) was approved (approval date 20 November 2013).

The time required for this study for each patient consisted of 3 months, which allows for observing the effects of the two non-surgical treatments in a proper timeframe.

At baseline (T = 0), the patients were instructed on adequate methods of oral hygiene, underlining the importance of tooth brushing and the use of interdental devices. In addition, 4 periodontal pockets with a probing pocket depth (PPD) of at least 5 mm were identified, dividing the patient’s mouth into 4 parts, with each part containing a periodontal pocket.

### 2.2. Randomization and Allocation Concealment

A split-mouth design was adopted. For each patient, the four eligible periodontal sites (one per quadrant; PPD ≥ 5 mm) were assigned to the four study arms (control, HBX ^x^1, HBX ^x^3, and O_3_) using a computer-generated randomization sequence (simple random allocation without restrictions) created prior to study start. Allocation was concealed by sequentially numbered, opaque, sealed envelopes prepared by a researcher not involved in clinical procedures or sample analysis. At the baseline visit (T0), immediately after site selection and before treatment delivery, the operator opened the next envelope to reveal the treatment assigned to each quadrant. The allocation list was not accessible to the laboratory personnel.

### 2.3. Blinding

Due to the nature of the interventions, the operator and participants were not blinded; however, laboratory analyses (mPCR/qPCR) were performed without knowledge of treatment allocation.

The treatment was performed under the same conditions after oral hygiene intervention (full mouth ultrasonic scaling and root planning (US-SRP):

− The control group (named control) received the oral hygiene intervention only at the first appointment;
− The group receiving the oral hygiene intervention, followed by the application of HBX one single time (named HBX ^x^1) at the first appointment;
− The group receiving the oral hygiene intervention, followed by the application of HBX three times (named HBX ^x^3), where the agent was applied for one single time for the first three appointments;
− The group receiving the oral hygiene intervention, followed by the application of O_3_ gas three times, where it was applied one single time for the first three appointments.

Specifically regarding the topical agents:

− The O_3_ gas was applied immediately after the oral hygiene intervention for 20 s (at a fixed concentration of 2100 ppm, with 80% of oxygen) three times, spaced out at one week from each other;
− The HBX was applied in the gingival sulcus for 30 s, then washed with physiological water [17,25].

Before each treatment, subgingival plaque bacteria were collected by using absorbent paper points, which were inserted into the gingival for 50 s and then placed in a sterile Eppendorf tube containing 1 mL of TE buffer (10 mM Tris-HCl, pH 8, 1 mM EDTA) for the next molecular investigation and stored at −20 °C until further processing [26]. A total of 4–5 tubes were collected at each sampling for each patient.

Each tube was uniquely labelled with the patient identification (ID), the treatment type and the time of the treatment.

Dental plaque sampling was carried out at the following times:

− T = 0: at baseline before the oral hygiene intervention;
− T = 1: one week after the first appointment and after the oral hygiene intervention at the appointment;
− T = 2: two weeks after the first appointment and after the oral hygiene intervention at the appointment;
− T = 3: one month after the first appointment;
− T = 4: three months after the first appointment.

The dental plaque samples collected from the periodontal pockets were used to detect, by two multiplex PCR (mPCR), different periodontal pathogen bacteria at different times and with different non-surgical treatments by HBX, once and three times, and O_3_ therapy, and compared with the control group (no treatment). The choice of these two modalities in the application of HBX was made to evaluate if the prolonged application of this desiccant agent could reach better and more consistent, efficient results.

**Figure 1:**
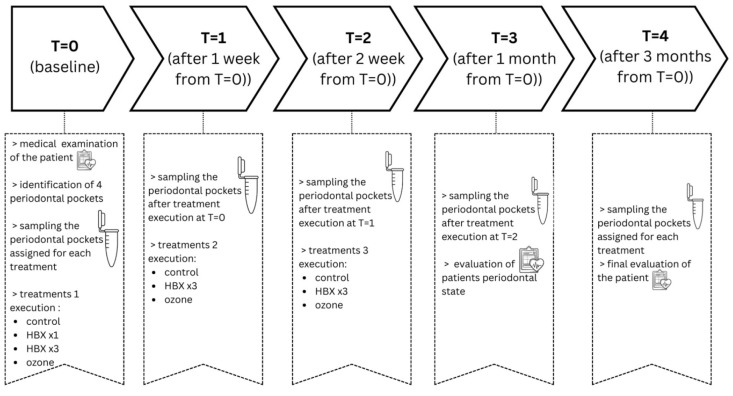
Flow-chart of this study.

The study flow-chart is available in Figure 1.

### 2.4. Microbiological Analysis

Microbiological analysis and molecular biology investigations were conducted by the Microbiology section of the Department of Diagnostics and Public Health at the University of Verona. The collected dental plaque was used to detect the presence of DNA from different pathogens by multiplex PCR (mPCR) and real-time quantitative PCR (qPCR) to detect and quantify *P. intermedia*. Briefly, genomic bacterial DNA from plaque was extracted using a GenElute™ Bacterial Genomic DNA Kit (Sigma-Aldrich, St. Louis, MO, USA), according to the manufacturer’s instructions, and the extracted DNA was stored at −20 °C until use. Two different multiplex PCRs (mPCR) were performed to identify the presence of *P. gingivalis*, *P. intermedia* and *A. actinomycetemcomitans* (mPCR-M1) [27], and *T. forsythia*, *T. denticola* and *A. naeslundii* (mPCR-M2) [28]. Additionally, an aliquot of genomic extract was stored at −20 °C to carry out, at a later time, the quantitative research on *P. intermedia* by qPCR. The first multiplex PCR (mPCR-M1) used a universal Forward primer 16s rRNA and a species-specific Reverse primer to detect the different microorganisms selected. In the second multiplex PCR (mPCR-M2), specific primer pairs for each pathogen were used. To evaluate the *P. intermedia* load, a quantitative real-time PCR (qPCR) was performed, using specific primers and probes [29], on a Fast Dx 7500 PCR system (Thermo Fisher Scientific, Waltham, MA, USA). PCR reactions were performed using 5Prime Hot Master Mix (Quantabio, Beverly, MA, USA), according to the manufacturer’s instructions. Primers, probes, and PCR conditions are reported in Table 1.

### 2.5. Outcomes

For the purpose of data interpretation, microbiological outcomes were defined a priori. The primary microbiological endpoint was the change in detection frequency of periodontal pathogens in subgingival plaque samples over time, following each treatment modality. Secondary endpoints included pathogen-specific detection patterns across treatments and time points, as well as the quantitative variation in *P. intermedia* assessed by qPCR between baseline (T0) and the end of follow-up (T4). Statistical analyses were conducted in accordance with these predefined outcomes.

### 2.6. Statistical Methods

For data collection, a database including all samples of patients evaluated in this study was created with Microsoft Excel. All data analysis was carried out using Stata v.13.0 for Macintosh (StataCorp, College Station, TX, USA). Microbiological qualitative data were expressed with absolute frequencies, percentages, and 95% confidence intervals. The comparison of these frequencies among groups was done using the Pearson Chi-square test; when values were less than 5, the Fisher exact test was used. The comparison of these frequencies at different times (T = 0, T = 1, T = 2, T = 3, and T = 4) was performed by using the Wilcoxon matched-pairs signed-rank test. The significance level was set at 0.05. When appropriate, the Bonferroni correction was applied for multiple comparisons between time points (5×).

With regard to microbiological variables, data were analyzed after the logarithmic transformation of the colony count data, with comparisons made among the mean values of the three groups using the Kruskal–Wallis test for non-Gaussian variables, and the non-parametric Wilcoxon test for unpaired data was employed to compare means at 2 different time points (T0 and T4).

## 3. Results

An initial screening was conducted on 50 patients. At the end of the screening phase, a total of 40 patients who met the inclusion criteria were enrolled in this study. As 10 patients declined to participate, 30 patients were finally available for this study, attending all follow-up visits, for a total of 120 samples (4 quadrants in the mouth for each patient). The enrolled patients were diagnosed with stage II or stage III periodontitis (grades A to C). Specifically, five patients presented stage II periodontitis and three patients stage III periodontitis, with four cases classified as grade A and four as grade B.

Demographics of the patients at T = 0 are available in Table 2.

**Figure 2:**
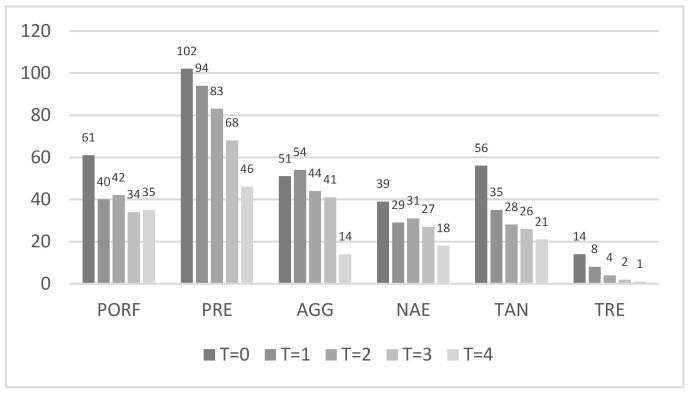
The distribution of periodontal pathogens detected in subgingival plaque samples from patients with periodontitis at baseline and after treatments. PORF = *Porphyromonas gingivalis*; PRE = *Prevotella intermedia*; AGG = *Aggregatibacter actinomycetemcomitans*; NAE = *Actinomyces naeslundii*; TAN = *Tannerella forsythia*; TRE = *Treponema denticola*. The numbers above each bar indicate the number of positive samples.

Qualitative data analysis showed that in the plaque samples analyzed, before and after the treatments, *P. intermedia* was the most frequently detected periodontal pathogen, while *T. denticola* was the one with the lowest detection rate (the value above the bar indicates the number of positive samples, see Figure 2).

The analysis of the data obtained from the two mPCRs (mPCR-M1 and mPCR-M2), considering individual periodontal pathogen bacteria, allowed us to highlight the following outcomes:

− For *P. gingivalis*, the O_3_ therapy showed a reduction in the detection, overall, at the last timepoints (T = 3 and T = 4). The desiccant agent (HBX) also showed a great reduction in microorganism presence when administered multiple times (HBX ×3) compared to that administered once. However, the comparison between groups at T4 was not statistically significant (*p* = 0.04, see Table 3).
− For *P. intermedia*, the HBX treatment showed similar results when applied multiple times or a single application to the periodontal pockets. Indeed, the data showed a reduction in the detection of microorganisms from the beginning (T0) to the end of the treatment (T4). The treatment with O_3_ also showed a reduction in microorganism detection at the end of the therapy. Comparison between groups at T4 was, however, not significant (*p* = 0.08, see Table 3).
− For *A. actinomycetemcomitans*, the HBX treatment applied once or three times showed comparable results in reducing this microorganism in the periodontal pockets. The O_3_ therapy was revealed to be more efficient in reducing the bacteria, especially in the long term. The comparison between groups at T4 was, in this case, significantly (*p* = 0.0002, see Table 3) in favor of the O_3_ treatment.
− For *A. naeslundii*, HBX treatment showed discordant results: in some cases, a single application showed a better efficiency in reducing the microorganism from the periodontal pocket, compared to the treatment repeated three times, and compared to the treatment with O_3_. Comparison between groups at T4 was, however, not significant (*p* = 0.15, see Table 3).
− For *T. forsythia*, the least detected microorganism, treatment with HBX administered three times with O_3_ ozone therapy was comparable and efficient over time in reducing the presence of the microorganism. Comparison between groups at T4 was, however, not significant (*p* = 0.08). HBX treatment applied three times with O_3_ therapy was effective in reducing the presence of this microorganism. Comparison between groups at T4 was, however, not significant (*p* = 0.11, see Table 3).

In summary, treatment with HBX three times shows efficacy in decreasing the load of all microorganisms tested, except for *A. naeslundii*, and O_3_ treatment showed similar efficacy compared to the control.

Further investigations were performed by qPCR to quantify the microbial load of *P. intermedia* in the plaque samples from the patients who were positive at time T = 0, and who were still positive (by mPCR) at time T4 (after 3 months), after the different treatments. To this end, a calibration curve of *P. intermedia* DNA was performed, using a serial dilution of the microorganism’s DNA [29]. In this way, it was possible to calculate the load of *P. intermedia* bacteria present in the plaque samples at the beginning and at the end of the different treatments. With the Ct value obtained by qPCR, the DNA concentration was calculated by the presence of the *P. intermedia* DNA, with a known concentration present in each run of the qPCR plate. Given the DNA concentration, the size (2.79 Mb) [30], and weight of the genome, it was possible to calculate the theoretical number of *P. intermedia* cells present in the plaque samples at the beginning and at the end of the different treatments, considering that each cell contains a single copy of the genome.

**Figure 3:**
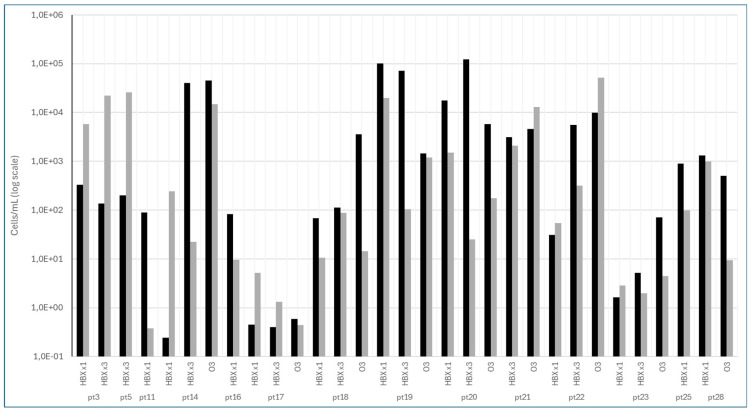
The qPCR results. The black bars indicate the *P. intermedia* bacterial load at baseline (T0), while the grey bars indicate the bacterial load at follow-up after treatment (T4). The y-axis shows the *P. intermedia* bacterial load. The x-axis reports individual patients (pts) grouped according to treatment: HBX ^x^1 (single application of HybenX), HBX ^x^3 (three applications of HybenX), and O_3_ (ozone therapy). Each assessment was performed in triplicate.

The qPCR results showed that after treatment with HBX ^x^1, *P. intermedia* DNA was detected in 12 out of 25 samples, and that in seven of these, a decrease in load was observed, while in five samples, an increase occurred. Treatment with HBX ^x^3 showed that in 10 out of 25 samples, the microorganism was still present, but in seven of these, the microbial load had decreased. Regarding O_3_, after treatment, nine out of 26 samples were still positive, and in seven of these, a decrease in *P. intermedia* load was observed (see Figure 3). Nevertheless, comparison between groups (HBX ^x^1, HBX ^x^3, and O_3_) and between time points (from T = 0 to T = 4) did not show any significant differences (*p* = 0.3 and *p* = 0.8).

## 4. Discussion

The integrity of the gingival epithelium can be violated by the presence of anaerobic Gram-negative bacteria present in the oral microbiota, which colonize the subgingival tissues, causing inflammation and leading to the formation of periodontal pockets [31]. If these pockets persist for a long time, they can cause tooth loss by increasing the resorption of the alveolar bone. Recent evidence has also highlighted how systemic inflammatory conditions and viral infections, such as coronavirus disease 2019 (COVID-19), may exacerbate oral inflammatory responses and periodontal tissue breakdown, further supporting the role of inflammation-driven mechanisms in periodontal disease progression [32].

In this study, we used multiplex PCR to evaluate the main periodontal pathogen bacteria belonging to the red (*P. gingivalis*, *T. forsythya*, and *T. denticola*) and orange (*P. intermedia*) Socransky complexes, along with *A. actinomycentemcomitans*, involved also in juvenile periodontitis, and *A. naeslundii*, associated with interactions with other oral microorganisms. By using two endpoint multiplex PCR reactions, we quickly and economically assessed the presence of six different periodontal pathogenic bacteria before and after various treatments.

However, it is known that endpoint PCR is only qualitative, meaning it provides information only in the presence or absence of the target pathogen’s genome. Therefore, we performed quantitative PCR on *P. intermedia* to assess whether the microbial load had changed in samples still positive after the various treatments. This approach allowed us to evaluate the changes in the microbial load of *P. intermedia* after the different treatments, indicating whether they were potentially effective. The choice to quantify *P. intermedia* by qPCR is due to its prevalence in periodontal sites, both before and after treatment, and also to its demonstrated relevance in periodontal disease.

In this study, we tested the activity of two non-surgical adjuvant treatments, HBX and O_3_ therapy, on oral microorganisms involved in periodontal diseases:

− HBX gel is a desiccant solution that can remove water molecules from biofilms, causing rapid dehydration and, consequently, disaggregation [33]. In this study, HBX was applied once at the first visit (T = 0) in one of the four periodontal pockets, while in another periodontal pocket, it was applied three times, 1 week apart (T = 0, T = 1) and then 1 month apart (T = 2). Using this method, the efficacy of oral microbiota bacteria was investigated after a single treatment and after three repeated applications [16].
− O_3_ consists of the use of gaseous ozone on the gingival margin, being a good disinfectant and an antimicrobial substance that is also able to stimulate tissue regeneration [20,34].

From the results obtained by multiplex PCR (mPCR-M1 and mPCR-M2), it was possible to deduce that the therapy with O_3_ showed the best and most efficient results from the microbiological point of view against *P. gingivalis* and *P. intermedia*, since, in the last timepoints (T = 3 and T = 4), a notable reduction in microbial load was observed.

Regarding HBX treatment, it showed better results in terms of the presence of microorganisms in the periodontal pocket when administered three times (HBX ^x^3) compared to the single application (HBX ^x^1).

For *A. actinomycetemcomitans*, the HBX treatment applied one or three times showed similar results in reducing these bacteria from the periodontal pockets. However, the O_3_ therapy was found to be more efficient in reducing it, especially in the long term after treatment.

For *T. forsythia*, the therapy with O_3_ seemed to be more efficient over time in terms of microbial reduction and the same results were observed by applying the HBX desiccant three times on the periodontal pocket.

For *T. denticola*, the least frequently detected pathogen, HBX treatment applied three times and O_3_ therapy were effective in reducing the presence of this microorganism. *T. denticola* is one of the most relevant bacteria involved in severe chronic periodontitis: the reduced presence of this bacterium could be related to the efficacy of the treatments, since it is observed in the literature that it colonizes the oral microbiota at a late stage of biofilm development [35].

For *A. naeslundii*, HBX treatment showed discordant data, as in some cases the single treatment reduced the presence of the microorganism more effectively than the repeated treatment. Also, regarding therapy with O_3,_ fluctuating results were observed, with an initial decrease, followed by an increased load at T2, then a reduction at T4. As *A. naeslundii* has been correlated with biofilm ecological balance and is frequently associated with a healthy microbiota, outcomes must be considered in this perspective. This species can indeed increase local pH by producing ammonia and alkali and can also metabolize lactic acid into weaker acids [36].

Since *P. intermedia* was the most frequently detected microorganism before and after the different treatments, and one of the main markers of gingivitis and periodontitis [37], we performed the quantification by qPCR in the samples that were positive to mPCR at the beginning (T = 0) and at the end of the different treatments (T = 4). From the evaluation of the microbial load of *P. intermedia* in the three different treatments, it emerged that, in most cases, even if the sample was still positive, the microbial load was reduced. This was overall evident in the treatment with O_3,_ where in seven out of nine samples positive at T4, a decrease in the microbial load was observed, despite a lack of statistical significance regarding comparisons between groups (HBX ^x^1, HBX ^x^3, and O_3_) and between time points (T = 0 and T = 4).

Although O_3_ appeared to be associated with a greater improvement in some clinical parameters, such as bleeding on probing and plaque index, these observations should be interpreted with caution. Clinical periodontal parameters were not quantitatively analyzed in this study, and therefore any potential clinical benefit should be considered exploratory and hypothesis-generating. The main strength of the present investigation lies in the microbiological evaluation of periodontal pathogens, which supports the antimicrobial effectiveness of ozone therapy as an adjuvant to non-surgical periodontal treatment.

We recognize that quantitative PCR for *P. intermedia* alone represents a limitation of this study, but this is based on both biological and practical considerations. *P. intermedia* was selected due to its high prevalence in our study population, both at baseline and after different non-surgical treatments. In contrast, other key periodontal pathogens, such as *P. gingivalis*, were detected less consistently in our biological sample.

The detection of *P. intermedia* alone cannot be considered fully representative of the effects, but our intention was to use *P. intermedia* as a potential indicator of treatment outcome.

For a more comprehensive investigation, we would have needed to quantify other microorganisms, but the limited sample size forced us to make choices, and we chose the most frequently detected periodontal pathogens. It is, thus, desirable to further explore this aspect in future studies using, for example, multispecies qPCR panels or broader approaches, such as sequencing, to further characterize the microbiological changes associated with different treatments.

However, it is possible to state that both tested therapies are effective in reducing the presence of the periodontal bacteria in pockets of patients affected by severe chronic periodontal diseases: HBX applied three times showed better results than a single application, while the O_3_ therapy showed similar and sometimes better results compared to HBX three times. Clinically, the O_3_ therapy showed a greater improvement in periodontal conditions, especially for the clinical parameters of bleeding on probing (reduction in bleeding on probing is considered a parameter to observe an improvement from the periodontal disease) and plaque index. The HBX therapy did not highlight differences, from a clinical point of view, compared to the control treatment of US-SRP.

From a treatment-oriented perspective, the overall microbiological findings suggest that both non-surgical adjuvant therapies were effective in reducing periodontal pathogens, although with partially different patterns. O_3_ therapy showed a more consistent and sustained reduction over time for several key pathogens, particularly *Porphyromonas gingivalis*, *Prevotella intermedia*, and *Aggregatibacter actinomycetemcomitans*, especially at the later follow-up time points. In contrast, the HBX desiccant agent demonstrated a treatment-frequency-dependent effect, with repeated applications (HBX ^x^3) generally resulting in greater microbial reduction compared to a single application, while showing more variable effects across different bacterial species. When considered across all the investigated microorganisms, O_3_ therapy appeared to provide a more homogeneous antimicrobial effect, whereas HBX efficacy was more closely related to the number of applications. These results are consistent with previous evidence on adjunctive non-surgical therapies, such as air polishing, which has been shown to improve microbial management in supportive periodontal treatment [38]. This complementary interpretation allows a more immediate comparison between the two treatment modalities and supports their role as adjuvant approaches in non-surgical periodontal therapy.

### Study Limitations

This study has several limitations that should be acknowledged.

First, the relatively small cohort of patients analyzed and the single-center University-based setting may limit the external validity of the findings.

Another limitation is that the quantitative analysis was performed only for *Prevotella intermedia*, which does not allow a comprehensive quantitative evaluation of all investigated periodontal pathogens.

Furthermore, the study population showed a marked gender imbalance, with a predominance of male patients, which may limit the generalizability of the results to a more gender-balanced population.

In addition, the cohort consisted predominantly of non-smoking patients; since smoking is a well-established risk factor for periodontal disease and is known to negatively influence treatment outcomes, this may have contributed to more favorable responses and limits the extrapolation of the results to smoking patients.

Therefore, a greater number of patients should be recruited to corroborate the outcomes, where each patient receives only one treatment, analyzing from a quantitative point of view more samples positive for *P. intermedia*. Furthermore, a planned quantification should be added by qPCR for the presence of other periodontopathogens, such as *P. gingivalis*, since it revealed synergism with *P. intermedia* in the development of oral pathogenic biofilm.

Regarding the split-mouth design used in this study, although effective in minimizing intersubject variability, it may present limitations in microbiological investigations. Specifically, it is not possible to completely rule out cross-contamination between quadrants through saliva, gingival crevicular fluid, or patient systemic responses. Cross-contamination could have influenced both the presence and bacterial load detected by mPCR and qPCR, potentially leading to an inaccurate estimate of the interventions’ effects. Therefore, microbiological results must be interpreted in conjunction with clinical data, and we hope, in the future, to make studies with parallel group designs to confirm these findings.

Finally, it could also be useful to investigate the possible synergistic effect of the combination of the two treatments, HBX and O_3_, on periodontal pockets.

## 5. Conclusions

The study described in this paper included 120 samples of dental plaque from 30 patients affected by periodontal disease, evaluating if the application of two non-surgical adjuvant therapies, HBX and O_3_, in addition to a professional oral hygiene intervention (US-SRP), could contribute to an improvement of clinical periodontal parameters, such as bleeding on probing and plaque index.

The effect of these treatments was evaluated from a microbiological point of view by detecting the presence of bacteria using the molecular biology techniques of multiplex PCR and qPCR. Specifically, regarding the qPCR of *P. intermedia*, it can be stated that the quantitative investigation only for *P. intermedia* represents a strong limitation, considering also the limited number of samples considered, for which the decrease in microbial load with treatment with O3 should be interpreted with caution.

Within the limitations of this study, both HBX and ozone therapy demonstrated effectiveness in reducing periodontal pathogens when used as adjuvant treatments to non-surgical periodontal therapy. The microbiological findings suggest a consistent antimicrobial effect of ozone therapy, while any observed clinical improvements should be interpreted as exploratory and require confirmation through studies specifically designed to quantitatively assess clinical periodontal outcomes.

## Figures and Tables

**Table 1:** Primers and PCR conditions used in this study.

Type	Target	Sequence	Amplicon Size Bp	Annealing Temp	PCR	Reference
F	16 s rRNA	5’CGTGCCAGCAGCCGCGGTAATACG3’		70 °C	mPCR-M1	García et al. 1998 [27]
R	*P. intermedia*	5’TCCGCATACGTTGCGTGCACTCAAG 3’	163			
R	*A.actinomycetemcomitans*	5’CTTTGCACATCAGCGTCAGTACATCCCCAAGG 3’	253			
R	*P. gingivalis*	5’TACATAGAAGCCCCGAAGGAAGACG 3’	527			
F	*T. denticola*	5’GCA AGA CTT GTA GCG GTA GT3’	890	60 °C	mPCR-M2	Carelli et al. 2023 [28]
R	*T. denticola*	5’ GAT GCC TAT TTG CGG GCT TG 3’				
F	*T. forsythia*	5’ CGG TGG TCT CCA ATC TCA CC 3’	559			
R	*T. forsythia*	5’ GCC CTC AAC ACA CGA CAC TT 3’				
F	*A. naeslundii*	5’ GGA ATG ATG GCG TGA ATG GC 3’	702			
R	*A. naeslundii*	5’ CCG ATC CCG TGA GTA CAT GG 3’				
PREq-F	*P. intermedia*	5’ TCC ACC GAT GAA TCT TTG GTC 3’			qPCR	Kuboniwa et al. 2004 [29]
PREq-R	*P. intermedia*	5’ ATC CAA CCT TCC CTC CAC TC 3’				
Probe	*P. intermedia*	5’ [FAM] CGT CAG ATG CCA TAT GTG GAC AAC ATC G [TAM] 3’				

F = primer Forward, R = primer Reverse, PREq-F: primer Forward qPCR, PREq-R: primer Reverse qPCR.

**Table 2:** Demographics at baseline. Variables related to patients are expressed as *n* (%); age is presented as mean ± standard deviation (SD).

Variable	*n* (%)
Gender	
male	23 (76.7)
female	7 (23.3)
Age	66 ± 4 years
Smoking	
no	28 (93.3)
yes (<10 cigarettes/day)	2 (6.7)
ASA (American Society of Anesthesiologists) status	
I	5 (16.67)
II	25 (83.3)
Periodontal diagnosis	
stage II grade A	26 (86.67)
stage III grade A	2 (6.67)
stage II grade B	1 (3.33)
stage III grade B	1 (3.33)

**Table 3:** DNA detection of the different microorganisms across the different treatments and times. The numbers in the table represent the positive DNA samples detected in the dental plaque samples for the different treatments (control, HBX ^x^1, HBX ^x^3, and O_3_) and at the different times (T1, T2, T3, and T4).

Bacteria DNA	Treatment	T0	T1	T2	T3	T4
*P. gingivalis*	Control	14	13	13	12	8
	HBX ^x^1	17	10	9	8	12
	HBX ^x^3	15	7	8	6	9
	O_3_	15	10	12	8	6
*P. intermedia*	Control	26	25	23	21	14
	HBX ^x^1	25	22	19	17	12
	HBX ^x^3	25	24	20	16	10
	O_3_	26	23	21	14	9
*A.actinomycetemcomitans*	Control	9	14	13	13	4
	HBX ^x^1	15	14	11	12	4
	HBX ^x^3	15	14	12	9	4
	O_3_	12	12	8	7	2
*A.naeslundii*	Control	13	13	11	4	4
	HBX ^x^1	12	4	4	7	4
	HBX ^x^3	8	7	10	12	7
	O_3_	6	5	6	4	3
*T. forsythia*	Control	13	8	10	9	5
	HBX ^x^1	12	7	6	7	6
	HBX ^x^3	13	8	5	5	5
	O_3_	18	12	7	5	5
*T. denticola*	Control	2	2	2	1	1
	HBX ^x^1	5	2	1	1	0
	HBX ^x^3	4	1	0	0	0
	O_3_	3	3	1	0	0

## Data Availability

Data are available from the corresponding authors upon reasonable requests.
